# PrtA-mediated flagellar turnover is essential for robust biofilm development in *Serratia marcescens*

**DOI:** 10.1128/aem.01261-25

**Published:** 2025-08-21

**Authors:** Marisel R. Tuttobene, Roberto E. Bruna, María Victoria Molino, Eleonora García Véscovi

**Affiliations:** 1 Instituto de Biología Molecular y Celular de Rosario, Consejo Nacional de Investigaciones Científicas y Tecnológicas, Universidad Nacional de Rosario 28237, Rosario, Santa Fe, Argentina; Indiana University Bloomington, Bloomington, Indiana, USA

**Keywords:** *Serratia*, biofilm, metalloprotease, PrtA, proteolytic activity

## Abstract

Biofilm formation is crucial for bacterial persistence, requiring precise regulatory mechanisms to transition from motility to sessility. Here, we uncover the role of the metalloprotease PrtA in *Serratia marcescens* biofilm development and its interaction with flagellar components. Loss of PrtA leads to reduced biofilm biomass, thickness, and viable cell counts, as shown through high-resolution confocal microscopy. The biofilm-deficient phenotype is rescued by wild-type PrtA expression but not by a proteolytically inactive PrtA_E177A_ mutant, underscoring the essential role of PrtA’s enzymatic activity. Exogenous addition of purified PrtA restores biofilm formation, confirming its enzymatic necessity. Proteomic profiling identified flagellar proteins as primary PrtA targets, with an overrepresentation of flagellar components in *prtA* mutant biofilms. In addition, PrtA selectively degrades depolymerized flagellar filaments, facilitating biofilm progression by removing excess flagellar material. Transcriptional analysis reveals an inverse expression pattern of flagellar master regulator (*flhDC*) and *prtA* during biofilm establishment, suggesting a coordinated regulatory axis that suppresses flagellar function while promoting biofilm development. Confocal microscopy at the liquid-air interface shows increased flagellar content in *prtA* mutant biofilms, supporting PrtA’s role in matrix organization and biofilm integrity. Collectively, these findings establish PrtA as a crucial mediator of flagellar turnover and extracellular proteolysis, linking motility suppression to robust biofilm formation. This work not only advances our understanding of biofilm regulation in *S. marcescens* but also identifies PrtA as a potential target for novel biofilm control strategies.

**IMPORTANCE:**

Biofilms are central to the persistence and pathogenicity of *Serratia marcescens*, particularly in clinical settings where they contribute to chronic infections and antimicrobial resistance. This study identifies the metalloprotease PrtA as a critical regulator of biofilm development, acting through the selective degradation of flagellar components to mediate the transition from motility to sessility. By demonstrating that PrtA’s proteolytic activity is essential for proper biofilm architecture and viability, and that it directly targets excess flagellar material, we provide mechanistic insight into how biofilm maturation is coordinated with motility suppression. The discovery of an inverse regulatory relationship between *prtA* and the flagellar master regulator *flhDC* further supports the existence of a finely tuned system controlling biofilm establishment. Together, these findings enhance our understanding of biofilm regulation in *Serratia marcescens*, an opportunistic human pathogen increasingly associated with antibiotic resistance, and highlight PrtA as a promising target for novel anti-biofilm strategies.

## INTRODUCTION

*Serratia marcescens* is a Gram-negative bacterial species that can be isolated from an ample range of environmental niches including soil, air, and water, and it can also colonize and infect plants and invertebrates (1). *S. marcescens* is also known as an emergent, opportunistic human pathogen, responsible for health-threatening diseases such as meningitis, infections of the urinary tract, corneal keratitis, pneumonia, and septicemia, and it is frequently identified as the source of nosocomial outbreaks (2). Because of the increasing occurrence of isolated multidrug-resistant strains (3), in 2017, the World Health Organization declared *Serratia*, along with other carbapenem-resistant Enterobacteriaceae, as a research priority to develop alternative antimicrobial strategies (4), this list was also updated in 2024 (5). The survival, persistence, and proliferation capacity of *S. marcescens* in such a wide variety of environmental locations and hosts relies on sensing and adaptation strategies where coordinated gene expression dictates the timely production of specific bacterial effectors.

The ability to attach and produce multicellular and structurally complex communities on biotic or abiotic surfaces confers bacteria several advantages to survive and thrive in challenging environments. These polymorphic communities, known as biofilms, allow bacteria to shield from antibiotic drugs, resist shearing forces and dehydration, counteract oxidative stressors and host-immune attacks among other harmful menaces (6). Biofilm formation also provides other beneficial traits to bacteria such as the sharing of common goods among cells and the production of locally concentrated extracellular enzymes that degrade complex nutrient sources or breach host defense barriers (7). Within biofilms, bacteria are embedded in a matrix formed by self-produced exopolysaccharides, extracellular DNA, enzymes, lipids, fimbria, flagella, and released metabolites (8).

The capacity of *Serratia* to form, survive, and persist in biofilms is a major healthcare, industrial, and ecological concern. These cell communities are highly refractory to be removed from nosocomial settings and indwelling biomedical devices (such as prostheses and catheters), they massively infect coral reef sanctuaries (9), and they also corrode industrial pipelines (10). Therefore, the identification of biochemical pathways and biological factors critical to biofilm formation is crucial to develop novel anti-bacterial strategies.

Factors that were found to be involved in the capacity of enterobacteria, including *Serratia*, to establish a biofilm comprise quorum-sensing communication strategies (11–14), cAMP intracellular levels (15), capsular polysaccharides (16), fimbria assembly (17), and biosurfactant production (18) as well as flagellar-mediated motility (19).

PrtA, also known as serralysin, serrapeptase or serratiopeptidase is one of the more abundant exoenzymes secreted by *Serratia*. PrtA belongs to the zinc-metalloproteases family of serralysins. The type I LipBCD secretion system exports PrtA to the extracellular medium (20–23). Our previous work demonstrated that *prtA* transcription is repressed by CpxR, the response regulator of the CpxAR two-component signaling system, whose activity was found to be induced at high temperatures. Accordingly, we showed that PrtA expression is transcriptionally activated at temperatures below 30°C and repressed at temperatures above 37°C. We also showed that the inactivation of PrtA expression in a *prtA* mutant strain was detrimental for the capacity of *Serratia* to form biofilm over an abiotic surface and, as expected, this phenotype was dependent on the growth temperature of the bacteria. Taken together, our findings suggested that PrtA was mainly involved in the life cycle of *Serratia* outside homeothermic mammalian hosts (22). However, the mechanism underlying the action of PrtA, which favors *S. marcescens* biofilm formation, was not completely elucidated.

PrtA has been explored as a potential useful molecule that might either prevent biofilm establishment or act as a biofilm dispersal agent of pathogenic bacteria other than *Serratia*. These properties were mostly attributed to the proteolytic activity of PrtA, which would inactivate factors required by bacteria to either attach to a surface or build the matrix in which the bacterial community is embedded (24). Because of this, PrtA was also proposed as an enhancer of antibiotic action that could be used to eradicate communities formed by bacteria other than *Serratia* (22, 25, 26). In line with these findings, the work by Selan and colleagues (27) showed that the *S. marcescens* ATCC 21074 PrtA homolog impaired the capacity of *Staphylococcus aureus* to attach to an abiotic matrix and develop biofilm. However, this phenotype was shown to be retained in a strain that expressed a single-amino acid PrtA mutant protein with an abrogated hydrolytic capacity. This observation opened the intriguing possibility that PrtA could be able to play a role in the remodeling of bacterial biofilm structure, with no involvement of its enzymatic activity.

In this work, we in depth examine the role of PrtA in *Serratia* biofilm formation. We demonstrate that, in contrast to the detrimental action on the biofilm formation of other bacteria, the expression of catalytically active PrtA is required as one important player in the consolidation of the biofilm three-dimensional architecture molded by *S. marcescens* on abiotic surfaces.

## MATERIALS AND METHODS

### Bacterial strains and plasmids

The strains and plasmids used in this study are listed in Table S1 (http://ibr-conicet.gov.ar/wp-content/uploads/2025/07/Tuttobene-et-al-Supplementary-Material.docx). The primers used in this study are listed in Table S2 (http://ibr-conicet.gov.ar/wp-content/uploads/2025/07/Tuttobene-et-al-Supplementary-Material.docx).

### Media and growth conditions

Strains were routinely cultured in Miller’s Luria-Bertani (LB) medium at the indicated temperature. For biofilm assays, SLB medium (peptone at 10 g/L and yeast extract at 5 g/L) was also used. The antibiotics used for selection in *E. coli* or *S. marcescens* were tetracycline, kanamycin, and ampicillin at concentrations of 4, 50, and 100 µg/mL, respectively.

### Genetic manipulations

To construct the pPrtA_E117A_ plasmid, primers prtA E177A Fw and prtA E177A Rv were used in a PCR using pPrtA as template. The resulting product was digested with *Dpn*I to eliminate parental methylated DNA and transformed into *E. coli* electrocompetent cells. E177A substitution was confirmed by Sanger sequencing. Subsequently, the pPrtA_E117A_ plasmid was mobilized into the *S. marcescens prtA* strain by conjugation.

*S. marcescens motA* was constructed as follows. PCR was used to generate 500 bp of DNA upstream of *motA* using primers BamHI UP *motA* FW and UP *motA* RV StuI (see Table S2 at http://ibr-conicet.gov.ar/wp-content/uploads/2025/07/Tuttobene-et-al-Supplementary-Material.docx) and ~500 bp of DNA downstream of *motA* using primers StuI DOWN *motA* Fw and DOWN *motA* RV XbaI (see Table S2 at http://ibr-conicet.gov.ar/wp-content/uploads/2025/07/Tuttobene-et-al-Supplementary-Material.docx). Through the splice by overlap extension (SOE)-PCR technique, both fragments served as primers for each other to generate a 1,000 bp product. The resulting DNA fragments were digested with the *BamH*I-*Stu*I restriction enzymes and ligated into the *BamH*I and *Stu*I sites of pKNG101 (28). pKNG101::*motA* recombinant plasmids, contents in the donor strain *E. coli* TOP10, were then mobilized into *S. marcescens* RM66262 by conjugation. Mutant strains were selected with streptomycin, and then high sucrose (15%, wt/vol) allowed the isolation of mutants in which the deletion allele had replaced the wild-type copy. The deletion of *motA* was confirmed by PCR using primers Ctrl *motA* FW and Ctrl *motA* RV (see Table S2 at http://ibr-conicet.gov.ar/wp-content/uploads/2025/07/Tuttobene-et-al-Supplementary-Material.docx).

Insertion mutation in *fliC* was constructed with the pKNOCK-Cm suicide plasmid (29). An internal 400 bp region was amplified using primers FliC mut Fw 300 *Xba*I and FliC mut Rv 700 *Xho*I (see Table S2 at http://ibr-conicet.gov.ar/wp-content/uploads/2025/07/Tuttobene-et-al-Supplementary-Material.docx). The purified PCR product was digested with the restriction enzymes indicated in the primer names and cloned into the pKNOCK-Cm plasmid. The resulting plasmids were introduced into competent *E. coli* SM10 λpir (30) cells by electroporation and then mobilized into *S. marcescens* RM66262 by conjugation. Insertional mutants were selected from chloramphenicol-resistant colonies, and chromosomal mutation was confirmed by PCR analysis.

To obtain the *slpE* mutant, the *slpE* gene along with its putative inhibitor *inhE* was first amplified by PCR using the plasmid pBB5::s*plE-inhE* as a template (lab stock) and the primers *slpE* ATG Fw (*Kpn*I) and *slpE inh* Rv (*Spe*I). The resulting PCR product was purified and digested with the restriction enzymes *EcoR*V and *Cla*I. The double digestion was run on a 1.5% agarose gel, and a 491 bp band corresponding to an internal region of the *slpE* gene was subsequently isolated and purified. This fragment was then ligated into the pKNOCK-Cm vector, previously digested with *EcoR*V and *Cla*I, generating the plasmid pKNOCK-Cm::*slpE*. The resulting plasmids were introduced into competent *E. coli* SM10 λpir (30) cells by electroporation and then mobilized into *S. marcescens* RM66262 by conjugation. Insertional mutants were selected from chloramphenicol-resistant colonies, and chromosomal mutation was confirmed by PCR analysis.

### Transcriptional expression level analyses

To analyze the transcriptional activity of *flhD*, the putative promoter region was PCR amplified from the chromosome using primers prom *flhD*-Fw and prom *flhD*-Rv (see Table S2 at http://ibr-conicet.gov.ar/wp-content/uploads/2025/07/Tuttobene-et-al-Supplementary-Material.docx) and cloned into pGEM-T. Afterward, pGEM-T::p*flhD* was digested with *EcoR*I enzyme, yielding a fragment which was subsequently cloned into the same site of pPROBE-NT [ASV] gfp-reporter vector (31). The resulting plasmids were introduced into competent *E. coli* Top10 cells by transformation. The plasmids P*flhD-gfp* was mobilized by conjugation into *S. marcescens*.

### Biofilm microscopy

Cultures of *S. marcescens* wild-type and *prtA* strains expressing GFP were grown with shaking overnight at 37°C. The bacterial cultures were washed with 1× phosphate-buffered saline (PBS). Next, 1/100 dilutions were made in SLB and were incubated in Nunc Lab-Tek II Chamber Slides at 30°C for 2, 3, and 6 days. Biofilms were examined using a Zeiss LSM880 confocal microscope scanning confocal laser microscope. Biofilm quantification was carried out using the COMSTAT analysis package as described (32, 33). The assay was repeated three times.

### *flhD* and *prtA* gene expression assays

Cultures of *S. marcescens* wild-type pSU36:mCherry P*prtA-gfp* and wild-type pSU36:mCherry P*flhD-gfp* strains were grown in SLB medium for 100 h at 30°C. Confocal fluorescence microscopy images were captured from the wild-type strain at 7, 28, 55, 79, and 100 h. Transcriptional activity was calculated as the ratio of GFP fluorescence and CHERRY fluorescence (IntDen GFP/IntDen CHERRY). The assay was repeated three times.

### Biofilm assay

The quantification of biofilm production was performed by following a previously established protocol (22), with slight modifications. In a 96-well microtiter plate, 2 µL of saturated cultures was inoculated into 200 µL of LB or SLB broth in sextuplicate and grown statically at the indicated temperature for 48 h. The culture was aspirated, and wells were washed with water. Each well was stained with 0.5% crystal violet for 15 min at room temperature and then washed three times with water. The wells were allowed to dry for 1 h before 200 µL of ethanol-acetone (80:20) was added, and the plate was shaken at room temperature for 1 h to dissolve crystal violet from the well walls. Finally, absorbance at 562 nm was determined using a Synergy 2 plate reader (Biotek). Results are expressed as percentages relative to the values obtained for the wild-type strain. Statistical significance was determined using a one-way analysis of variance (ANOVA) followed by Tukey’s multiple comparison test. Asterisks indicate the significance levels for the statistical analysis: ∗, *P* < 0.05; ∗∗, *P* < 0.01; ∗∗∗, *P* < 0.001; and ∗∗∗∗, *P* < 0.0001, the analysis was performed using GraphPad Prism (GraphPad Software, San Diego, CA, USA). *P* < 0.05 was considered significant.

To determine the number of colonies forming units (CFU) forming the biofilm, cells were scraped off and passed several times through a tip and vortexed to disrupt cell clumps and obtain single cells. Biofilm cells were plated out in the corresponding antibiotic. The assay was repeated three times.

### Adhesion assays

Saturated cultures of *S. marcescens* strains were grown, diluted to obtain an OD600 of 1 in 200 µL, and grown in SLB culture medium at 30°C for 2 h in 96-well microtiter plates. The adhered bacterial or biofilm mass was measured by crystal violet staining as described in the previous protocol. Results are expressed as percentages relative to the values obtained for the wild-type strain. Statistical significance was determined using a one-way analysis of variance (ANOVA) followed by Tukey’s multiple comparison test. Asterisks indicate the significance levels for the statistical analysis: ∗, *P* < 0.05; ∗∗, *P* < 0.01; ∗∗∗, *P* < 0.001; and ∗∗∗∗, *P* < 0.0001; the analysis was performed using GraphPad Prism (GraphPad Software, San Diego, CA, USA). *P* < 0.05 was considered significant. The assay was repeated three times.

### Indirect immunofluorescence and fluorescence microscopy

Sterile coverslips were placed on the bottom of 24-well microplates at a 45° angle. Cultures were then applied slowly to cover half of the coverslips. The cultures were then cultured for 48 h in SLB culture medium at 30°C, protected from light. The coverslips were then carefully washed with PBS, and the biofilms were fixed with 3% paraformaldehyde for 15 min. The coverslips were subsequently washed with PBS and incubated for 1 h with the polyclonal anti-flagellin primary antibody (1:100) at room temperature. Three washes with PBS were then performed, and the biofilms were incubated for 1 h with Cy3-conjugated anti-rabbit secondary antibodies (1:150). The coverslips were then washed again with PBS and mounted with the Slow Fade Antifade reagent in glycerol/PBS. Flagella abundance was calculated as a ratio of Cy3 fluorescence and GFP fluorescence (IntDen Cy3/IntDen GFP) of the images obtained. Three independent experiments were done.

### Growth of *S. marcescens*

To test the ability of the *S. marcescens* wild type (wt), wt/pGFP, *prtA,* and *prtA*/pGFP to grow at 30°C without agitation, 1/100 dilutions of overnight cultures grown in SLB were inoculated in fresh SLB and grown in 96-well microplates at 30°C without agitation. OD600 nm was determined, every hour, for 20 h. The assay was repeated three times.

### Protease assays

For quantitative analysis, protease activity (azocaseinase assay) was measured from culture supernatants using azocasein (Sigma) as a colorimetric substrate as previously described (22, 34). Cultures were centrifuged and filtered to remove bacteria. A 50 µL aliquot of the filtered supernatant was mixed with 50 µL of 1% (wt/vol) azocasein and 140 µL of PBS and incubated for 1 h at 37°C. The reaction was stopped by addition of 80 µL of 10% (vol/vol) trichloroacetic acid, and the mixture was incubated for 15 min on ice. The tubes were centrifuged at 10,000 *g* for 10 min. The clear supernatant was removed, and its absorbance at 340 nm (A340) relative to that of a medium control was determined. This value was then normalized to the optical density at 600 nm (OD600) from the original culture.

### Proteomic analysis

*S. marcescens* wild-type and *prtA* were cultured without aeration in SLB, at 30°C for 48 h, in microcentrifuge tubes. Extracellular and biofilm matrix-associated cell fractions were recovered from both strains’ bio-pellicles. The supernatant was filtered through a 0.2 µm membrane to eliminate potential carryover of planktonic cells and cell debris and retain only soluble components. In parallel, the adhered cells and matrix attached to the surface of the tubes were also recovered. It was performed in triplicate. The samples of interest were submitted to the CEQUIBIEM proteomic facility in Argentina. A Thermo Scientific Q-Exactive spectrometer was used. The equipment features a High Collision Dissociation (HCD) cell and an Orbitrap analyzer. The equipment configuration allows peptide identification to be performed simultaneously with chromatographic separation, resulting in full MS and MSMS. Quantification was performed by calculating the areas for each protein. These areas were calculated according to the algorithms used by the Proteome Discoverer program. Finally, the data obtained were processed using the Perseus program. Database (source): *Serratia marcescens* subsp. marcescens UP000050507. Proteins showing a fold change (FC) >1 between *prtA* and wild-type strains and *P* value < 0.05 were considered differentially expressed.

### PrtA purification

The purification was performed by following the protocol of Belas et al. (35) with slight modifications. The PrtA protease was purified by phenyl-Sepharose hydrophobic interaction chromatography. Briefly, *S. marcescens slpE* was incubated overnight at 30°C in 1 L cultures of SLB broth. Cells and debris were removed by centrifugation at 10,000 × *g* for 30 min at 4°C. The protease-containing supernatants were then filtered through 0.45-mm-pore-size filters (Millipore). Supernatant was applied on a Q sepharose XL column and eluted with PBS + 2 M NaCl pH 7.4 using an Akta (GE Healthcare). Active fractions were pooled and concentrated by lyophilization and resuspended in PBS before gel filtration on a Superdex 75 column (GE Healthcare). The purity of the PrtA was determined by sodium dodecyl sulfate-polyacrylamide gel electrophoresis (SDS-PAGE).

### Flagella extraction

Flagella were isolated from the *prtA* strain as described (36). Cells were grown overnight in SLB at 30°C, and bacteria were pelleted by centrifugation. Pellets were resuspended in PBS, and flagella were sheared from bacteria by blending, followed by centrifugation at 8,000 × *g* for 15 min to pellet the bacteria. Flagella were collected from the supernatant by centrifugation at 100,000 × *g* for 60 min. The pellets obtained were resuspended in PBS, and purity was examined by SDS-PAGE. Isolated flagella were heated at 70°C for 20 min for depolymerization. Polymeric and monomeric flagellin was incubated with 0, 1, 2, 3, 6, and 10 µg/mL recombinant PrtA for 1 h at 37°C and analyzed by SDS-PAGE (36).

## RESULTS

### Enzymatically active, secreted PrtA contributes to *Serratia* biofilm formation

We have previously demonstrated that a *S. marcescens prtA* mutant, unable to express the PrtA metalloprotease, showed a diminished ability to form biofilm either at 30°C or 37°C, when bacteria were grown in LB or SLB media. Under all conditions assayed, the *prtA* defective biofilm phenotype was restored to wild-type levels by ectopic expression of PrtA (22).

**Fig 1:**
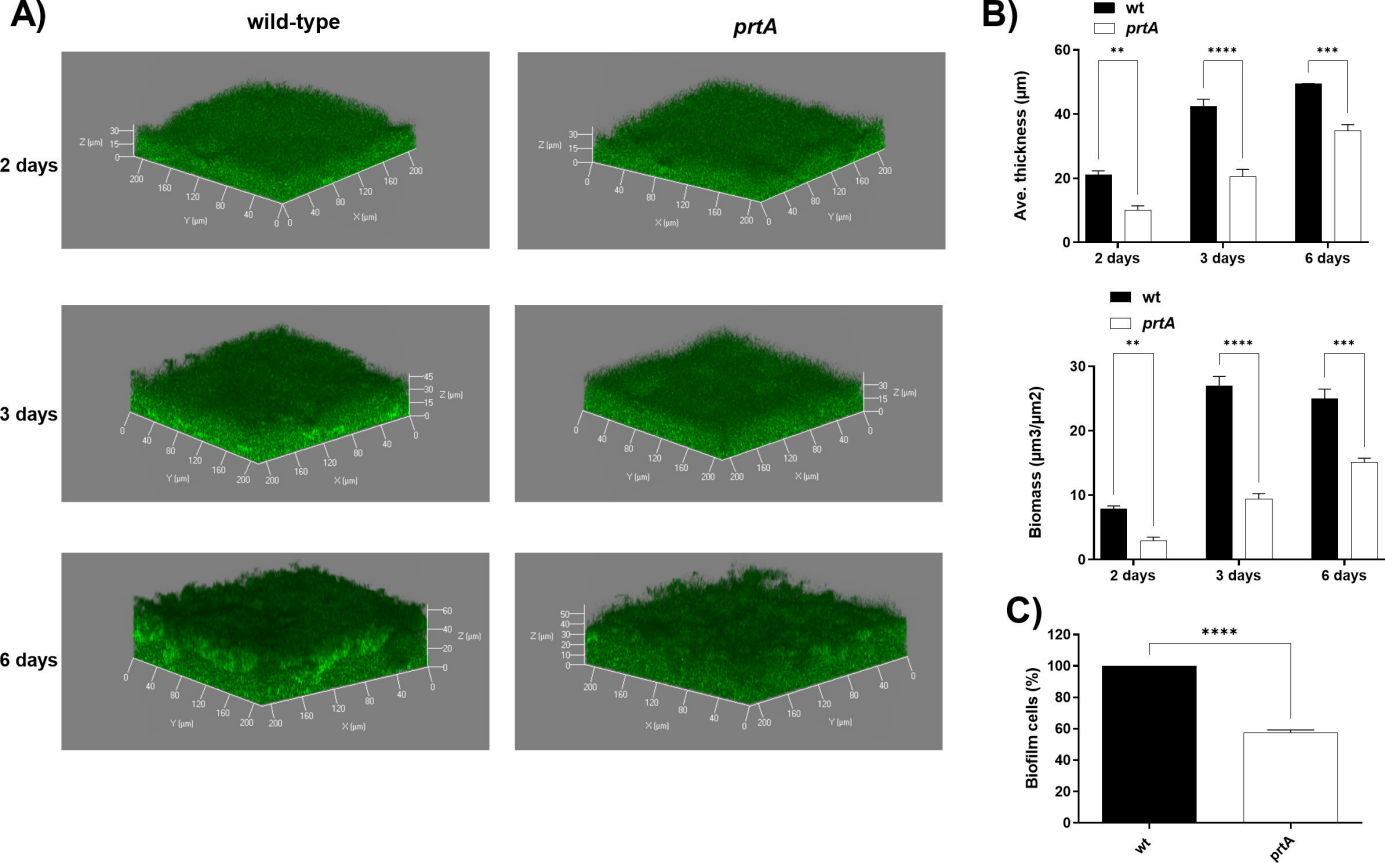
Confocal fluorescence microscopy images of wild-type and *prtA* strains expressing GFP in static biofilms grown in microtiter plates at 30°C for 2, 3, and 6 days, in SLB medium. (**A**) 3D reconstructions of biofilm images, generated with ZEN software. (**B**) COMSTAT results quantifying the average thickness (µM) and total biomass (µM3/µM2) of confocal microscopy images. (**C**) Strains were grown in SLB culture medium at 30°C for 48 h in 96-well microtiter plates. Cells were scraped off and passed several times through a tip and vortexed to disrupt cell clumps and obtain single cells. Biofilm cells were plated out to determine the number of colonies forming units (CFU). Results are expressed as percentages relative to the values obtained for the wild-type strain. Means ± SDs from three independent experiments performed in duplicate in each case are shown. Significant differences between strains calculated by unpaired *t* test are indicated as follows: *P* < 0.05; ∗∗, *P* < 0.01; ∗∗∗, *P* < 0.001; and ∗∗∗∗, *P* < 0.0001; the analysis was performed using GraphPad Prism (GraphPad Software, San Diego, CA, USA).

To gain insight into the contribution of PrtA to *S. marcescens* biofilm formation, we examined the structural features of the biopellicle. We analyzed biofilm formation by means of confocal microscopy 3-D images followed by calculation of structure parameters by COMSTAT analysis (32, 33, 37). For this purpose, we transformed wild-type and *prtA* mutant strains with a plasmid that constitutively expresses the green fluorescence protein (pGFP). Bacteria were statically grown in polystyrene cuvettes in SLB medium at 30°C. Image stacks were collected after 2, 3, and 6 days of incubation (Fig. 1A). The *prtA* strain showed a reduction of 50% of both total biomass and average thickness compared to the levels reached by the wild-type strain (Fig. 1B). This correlated with a 50% reduction of viable cell count values from *prtA* mutant biofilm samples in comparison to the wild-type, determined at 48 h post-inoculation. (Fig. 1C). As control, we verified that GFP constitutive expression altered neither bacterial growth (see Fig. S1A at http://ibr-conicet.gov.ar/wp-content/uploads/2025/07/Tuttobene-et-al-Supplementary-Material.docx) nor the biofilm capacity of these strains when grown at 30°C for 48 h in 96-well microtiter plates (see Fig. S1B at http://ibr-conicet.gov.ar/wp-content/uploads/2025/07/Tuttobene-et-al-Supplementary-Material.docx).

**Fig 2:**
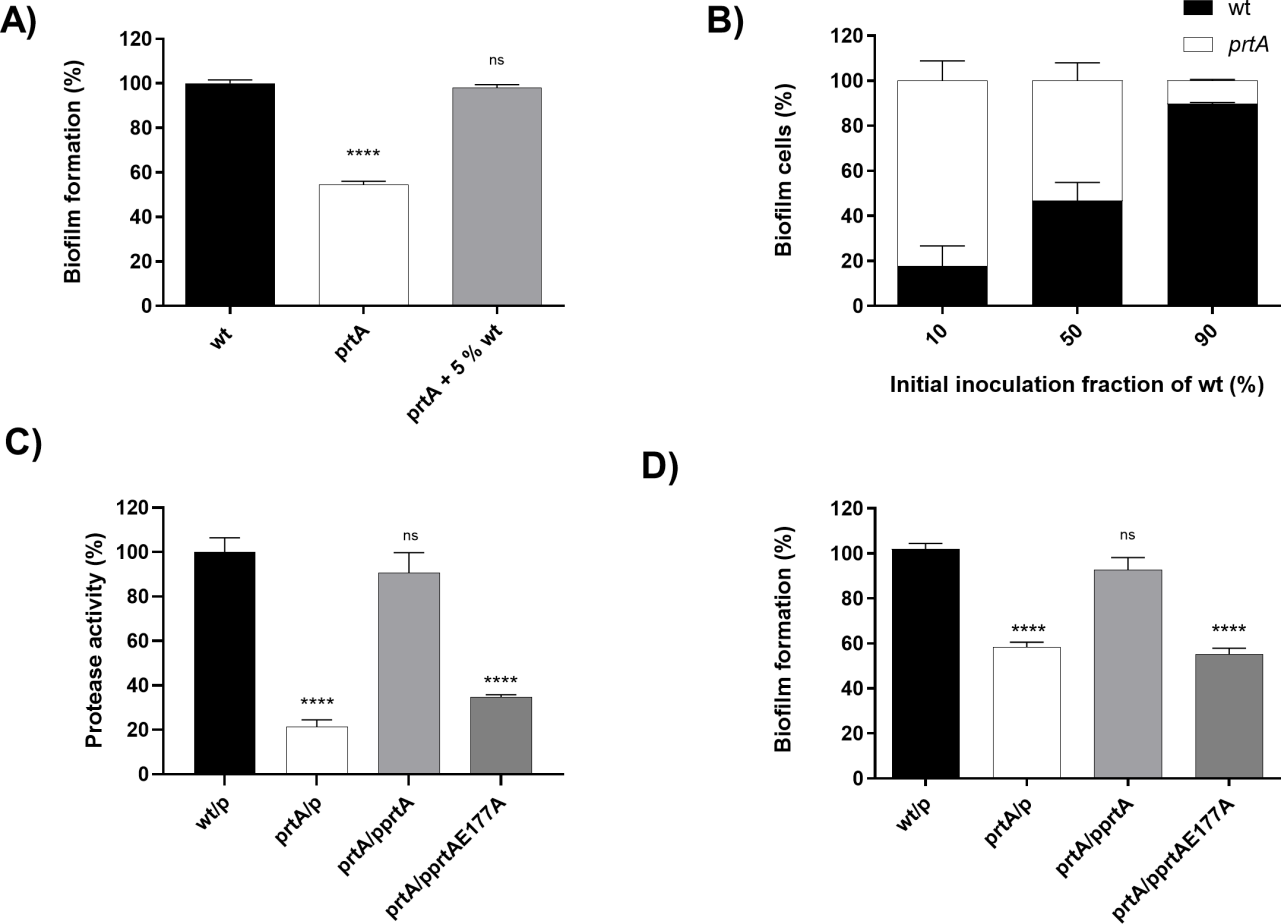
Biofilm complementation assay of the *prtA* mutant with the wild-type strain, and catalytically active and inactive *prtA*. (**A**) Cultures of the wild-type and *prtA* strains were co-inoculated in SLB culture medium, including unmixed wild-type and *prtA* strains. The lowest percentage of wild-type inoculum (5%) is shown, as higher percentages produced similar saturated complementation effects. Cultures were incubated for 48 h at 30°C in 96-well polystyrene microplates. The adhered biofilm was measured by crystal violet staining. Results are expressed as percentages relative to the values obtained for the wild-type strain. (**B**) Cultures of the wild-type and *prtA* strains were co-inoculated at indicated percentages in SLB culture medium and cultured for 48 h at 30°C in 96-well polystyrene microplates. Cells were scraped off and passed several times through a tip and vortexed to disrupt cell clumps and obtain single cells. Biofilm cells were plated out in the corresponding antibiotic to determine the number of colonies forming units (CFU). (**C**) Protease activity quantitative by azocasein assay in *S. marcescens* RM66262 wild-type harboring the empty plasmid (wt/p), *prtA* harboring the empty plasmid (*prtA*/p), *prtA*/p*prtA* and *prtA*/p*prtA*_E177A_. Results are expressed as percentages relative to the values obtained for the wild-type strain. (**D**) Strains were grown in SLB culture medium at 30°C for 48 h in 96-well microtiter plates. The adhered biofilm was measured by crystal violet staining. Results are expressed as percentages relative to the values obtained for the wild-type strain. Means ± SDs from three independent experiments are shown. Statistical significance was determined using a one-way analysis of variance (ANOVA) followed by Tukey’s multiple comparison test. Asterisks indicate the significance levels for the statistical analysis: ∗, *P* < 0.05; ∗∗, *P* < 0.01; ∗∗∗, *P* < 0.001; and ∗∗∗∗, *P* < 0.0001; the analysis was performed using GraphPad Prism (GraphPad Software, San Diego, CA, USA). *P* < 0.05 was considered significant. p: pBB2.

If secreted PrtA contributes to biofilm maturation, then we hypothesized that PrtA from wild-type cells would rescue the biofilm defect of the *prtA* mutant. To test this, wild-type and *prtA* strains were co-cultivated in different ratios (ranging from 5% to 90% wild-type cells) in polystyrene microwell plates, and the biofilm biomass was monitored by the crystal violet (CV) assay (Fig. 2A). We also determined the percentage of CFUs of each strain within the mixed biofilm to discard differential viability (Fig. 2B). Remarkably, even the lowest proportion tested (5% wild-type cells, the only percentage shown in Fig. 2A) was sufficient to complement the biofilm defect of the *prtA* mutant, reaching biofilm levels similar to those of the wild-type strain; higher proportions produced comparable results, indicating a saturating effect. The initial CFUs ratios between the two strains were maintained during 48 h, showing that there was no prevalence of one strain over the other one within the mixed biofilm development during this time frame, as long as one of the two strains expresses PrtA (Fig. 2A and B).

To address whether enzymatic activity of PrtA is required for biofilm maturation, we constructed the pPrtAE177A expression vector, which harbors a *prtA* mutant gene that encodes PrtA with a single amino acid substitution of glutamic acid by alanine in the zinc binding consensus HEXXHXXGXXH (27). This mutation completely abrogated proteolytic activity. The protease activity of PrtA and PrtAE177A secreted proteins was quantitated by the azocaseinase assay (Fig. 2C). While *in trans* expression of pPrtA restored the biofilm defective phenotype of the *prtA* mutant strain, pPrtA_E177A_ was not able to complement the defective phenotype (Fig. 2D).

**Fig 3:**
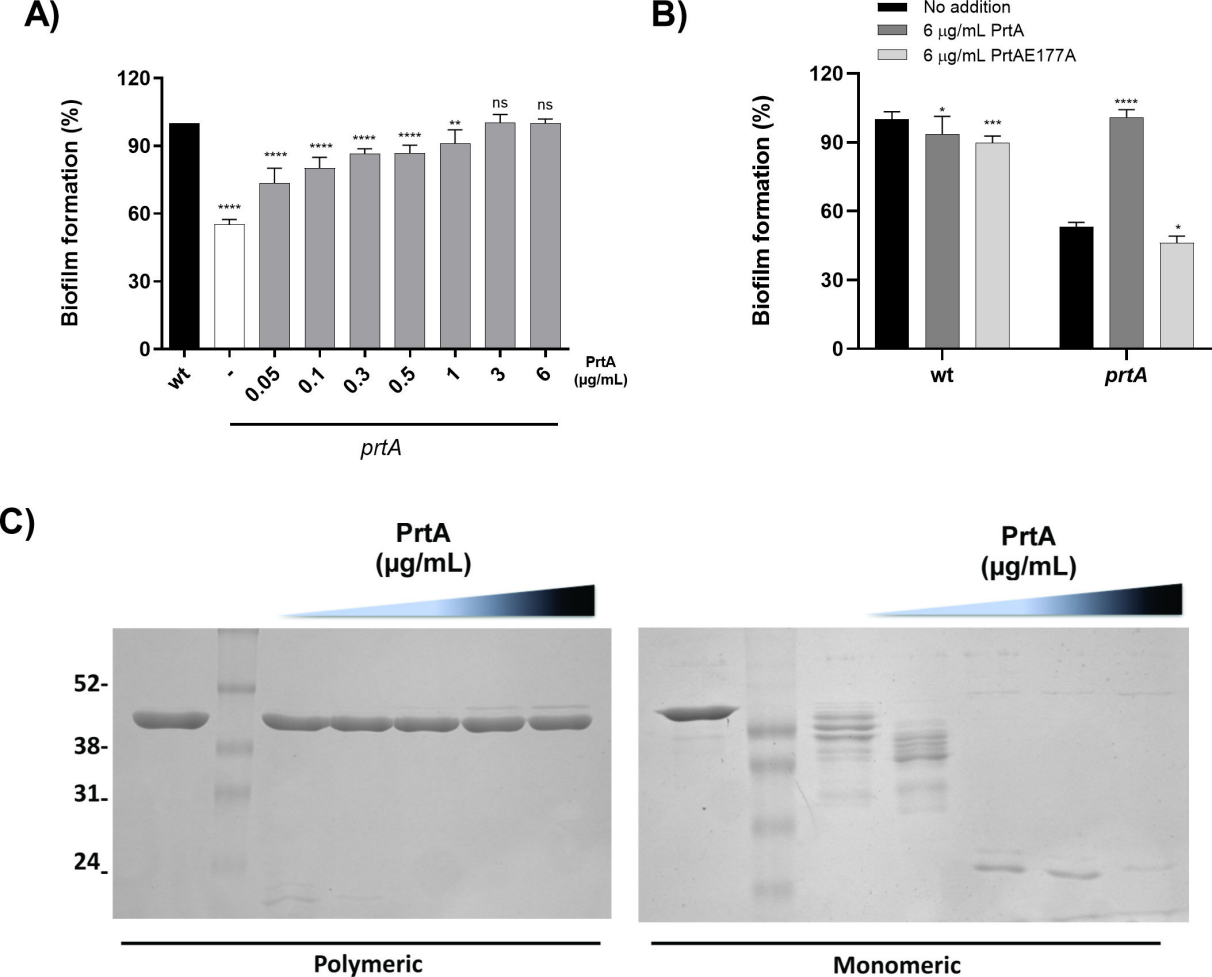
PrtA degrades only monomeric flagellin, while the integrity of flagella is preserved. (**A**) Strains were grown in SLB culture medium at 30°C for 48 h in 96-well microtiter plates. *prtA* strain was supplemented with indicated concentrations of purified PrtA, catalytically active, at the beginning of the assay. The adhered biofilm was measured by crystal violet staining. Results are expressed as percentages relative to the values obtained for the wild-type strain. Means ± SDs from three independent experiments are shown. (**B**) Strains were grown in SLB culture medium at 30°C for 48 h in 96-well microtiter plates. wild-type and *prtA* strains were supplemented with purified PrtA, catalytically active (PrtA) and inactive (PrtA_E177A_), at the beginning of the assay. The adhered biofilm was measured by crystal violet staining. Results are expressed as percentages relative to the values obtained for the wild-type strain without protease supplementation. Means ± SDs from three independent experiments are shown. Statistical significance was determined using a one-way analysis of variance (ANOVA) followed by Tukey’s multiple comparison test. Asterisks indicate the significance levels for the statistical analysis: ∗, *P* < 0.05; ∗∗, *P* < 0.01; ∗∗∗, *P* < 0.001; and ∗∗∗∗, *P* < 0.0001; the analysis was performed using GraphPad Prism (GraphPad Software, San Diego, CA, USA). *P* < 0.05 was considered significant. (**C**) Flagella isolated from *prtA* strain were treated for 20 min at 70°C to obtain monomeric flagellin. Untreated polymeric flagellin was compared with monomeric flagellin for susceptibility to PrtA cleavage. Polymeric and monomeric flagellin was incubated with 0, 1, 2, 3, 6, and 10 µg/mL recombinant PrtA for 1 h at 37°C and analyzed by SDS-PAGE.

To assess that isolated PrtA is able to exert its action, the *prtA* strain was grown in the absence or the presence of increasing concentrations of soluble purified PrtA protein. The biofilm mass was determined by the crystal violet staining assay after 48 h. The addition of a final concentration of 3 µg/mL of PrtA protein to *prtA* was sufficient for this mutant strain to reach the same biofilm mass levels as those developed by an equivalent initial inoculum of the wild-type strain alone (Fig. 3A). In contrast, the addition of purified PrtA_E177A_ to the *prtA* strain did not alter the levels of biofilm mass reached by the mutant strain alone (Fig. 3B). Altogether, these results demonstrate that the secreted, catalytically active, form of PrtA has a role in building the structure of *S. marcescens* biofilm.

### Flagellar components are the main substrate of PrtA

In light of the results shown above, we could think of two different scenarios: (i) PrtA timely degrades a substrate that is detrimental for biofilm formation turning it into an inactive molecule, or (ii) PrtA action converts an otherwise inert substrate into a molecule that favors biofilm formation.

To explore potential PrtA substrates within the biofilm, we performed a proteomic analysis by LC-MS/MS to either extracellular (supernatant) and biofilm matrix-associated cell fractions recovered from bio-pellicles of the wild-type and *prtA* mutant strains. For this purpose, we cultured bacteria without aeration in SLB, at 30°C for 48 h, in microcentrifuge tubes. We collected the supernatant and filtered this fraction through a 0.2 µm membrane to eliminate potential carryover of either planktonic cell on cell debris and to retain only soluble components. In parallel, the adhered cells and matrix attached to the surface of the tubes were also recovered.

Proteomic results are shown in Table S3 (http://ibr-conicet.gov.ar/wp-content/uploads/2025/07/Table-S3-2025.xlsx). A total of 36 and 83 proteins significantly overrepresented (fold change ≥1; *P* < 0.05) in the *prtA* mutant relative to the wild-type strain in the membrane and supernatant fractions, respectively. Among the membrane-associated proteins, we identified those involved in outer membrane integrity (e.g., OmpX, fold change = 4.5, *P* = 0.028), stress response (e.g., universal stress protein UspA, fold change = 2.3, *P* = 0.025), and biofilm regulation (e.g., BssS, fold change = 4.07, *P* = 0.0003). In contrast, the supernatant fraction was enriched in structural components of the flagellum, including FliC (fold change = 5.26, *P* = 0.016) and FliD (fold change = 5.56, *P* = 0.001), suggesting that these proteins may be released as a result of flagellar turnover and represent key substrates of PrtA activity. Differentially abundant proteins in both fractions also included various membrane transporters and enzymes implicated in redox balance and metabolic adaptation.

These findings support a model in which PrtA modulates the biofilm environment by degrading specific extracellular and membrane-associated proteins. Notably, flagellar subunits were the most enriched protein category in the *prtA* mutant, both in the supernatant and in the matrix-attached fractions, suggesting that these structures are key targets of PrtA proteolytic activity. Table 1 highlights selected flagellar proteins with their corresponding fold-change values between *prtA* and wild-type strains across the different biofilm fractions. Collectively, these results indicate that the building blocks of the flagellar appendage are the main targets of PrtA proteolytic activity, either attached to surface-adhered bacterial cells within the biofilm structure or as part of the spent supernatant. These findings strongly suggest that PrtA degrades flagellar subunits released during turnover and that this activity enhances biofilm formation. Because the lack of flagellar appendages strongly precludes the initial attachment biofilm formation, by using a flagellar mutant strain it would be not possible to evaluate the beneficious effect of their absence within the biofilm matrix at late stages of biofilm formation.

**TABLE 1:** Flagellar apparatus proteins detected in the matrix and supernatant of the *S. marcescens* RM66262 biofilm, which are exclusively or significantly represented in the *prtA* mutant compared to the wild-type strain

	Protein	Reference sequence in NCBI	Fold change (*prtA*/wt)
Membrane/ matrix	FlgI	WP_004934816.1	Only present in *prtA*
	FlgH	WP_004934817.1	Only present in *prtA*
	FliC	WP_033653649.1	+2.8
	FliD	WP_033647758.1	Only present in *prtA*
Supernatant	FlgK	WP_039565509.1	Only present in *prtA*
	FlgD	WP_033634994.1	Only present in *prtA*
	FlgL	WP_033634987.1	Only present in *prtA*
	FliC	WP_033653649.1	+5.26
	FliD	WP_033647758.1	+5.56

Bardoel et al. previously showed that AprA, a *P. aeruginosa* metalloprotease from the serralysin family, that shares 54% amino acid identity with PrtA, can degrade depolymerized flagella but not flagellar filaments (36). We purified both intact (polymeric) and monomeric *S. marcescens* flagellar filaments (as described in Materials and Methods [36]). Both preparations were co-incubated with increasing concentrations of purified PrtA up to 10 µg/mL, and the products were analyzed by SDS-PAGE. PrtA, similar to AprA, was only able to degrade depolymerized flagellar filaments (Fig. 3C). In sum, these results indicate that the proteolytic action of PrtA targets proteins released by bacteria as the result of flagellar protein turn-over. They also suggest that flagellar components would be detrimental for the progression in the formation of the *Serratia* biofilm structure.

**Fig 4:**
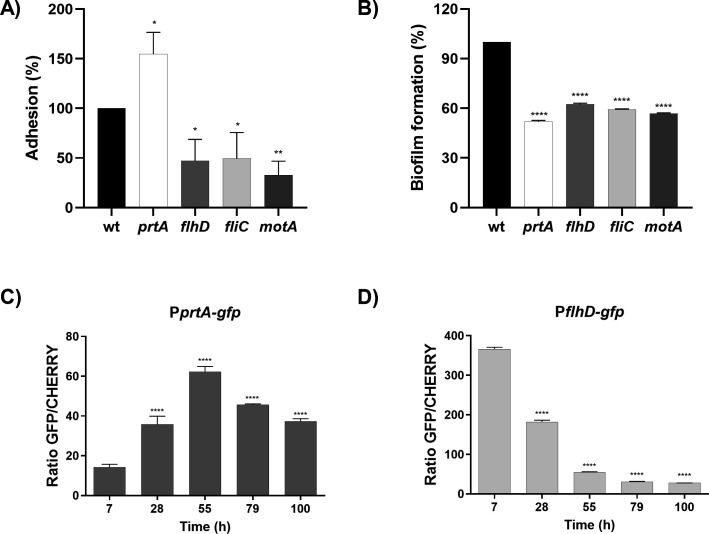
Adhesion, biofilm, and evaluation of transcriptional expression. (**A**) Adhesion assay. Saturated cultures of both strains were grown, diluted to obtain an OD600 of 1 and grown in SLB culture medium at 30°C for 2 h in 96-well microtiter plates. The adhered bacterial mass was measured by crystal violet staining. Results are expressed as percentages relative to the values obtained for the wild-type strain. Means ± SDs from three independent experiments are shown. (**B**) Biofilm assay. Strains were grown in SLB culture medium at 30°C for 48 h in 96-well microtiter plates. The biofilm mass was measured by crystal violet staining. Results are expressed as percentages relative to the values obtained for the wild-type strain. (**C and D**) Evaluation of transcriptional expression of *flhD* (**C**) and *prtA* (**D**) during biofilm formation. Strains were grown in SLB medium for 100 h at 30°C. Confocal fluorescence microscopy images were captured from the wild-type strain carrying the pSU36:mCherry and the P*prtA-gfp* or P*flhD-gfp* reporter plasmids at the indicated times. Transcriptional activity was calculated as the ratio of GFP fluorescence and CHERRY fluorescence (IntDen GFP/IntDen CHERRY). Means ± SDs from three independent experiments are shown. Statistical significance was determined using a one-way analysis of variance (ANOVA) followed by Tukey’s multiple comparison test. Asterisks indicate the significance levels for the statistical analysis: ∗, *P* < 0.05; ∗∗, *P* < 0.01; ∗∗∗, *P* < 0.001; and ∗∗∗∗, *P* < 0.0001; the analysis was performed using GraphPad Prism (GraphPad Software, San Diego, CA, USA). *P* < 0.05 was considered significant.

The first stage of biofilm formation is the bacterial attachment to a surface. The flagellar appendage is known to be required for the attachment of several enterobacteria to diverse surfaces (38–40). In fact, it is widely accepted that motility enhances bacterial aggregation by increasing collision events in viscous environments (41). Therefore, we investigated the interplay of PrtA and flagella along biofilm development. At earlier times, our results show that the *prtA* strain achieves higher levels of attachment than the wild-type strain indicating that adhesion to the abiotic surface is negatively influenced by PrtA expression (Fig. 4A). At 48 h post-inoculation, the biofilm mass achieved by the *prtA* strain equaled the levels of *fhlD* (devoid of flagella), *fliC* (unable to generate the flagellar filament structure), or *motA* (with intact, non-motile flagella) flagellar mutant strains (Fig. 4B). This clearly indicates that unlike the flagellar appendix, PrtA expression benefits biofilm growth at later stages beyond attachment. Taken together our results lead us to conjecture that PrtA contributes to modulate flagellar-mediated biofilm phases.

### Dynamics of PrtA and flagellar transcriptional expression

We next investigated whether PrtA degradation of flagellar components during the maturation of *Serratia* biofilm is accompanied by changes in gene expression at the transcriptional level. Upon bacterial inoculation on the abiotic surface, we monitored the transcriptional activity of *flhDC*, which encodes for the flagellar master regulator (42–44), and of *prtA*. The promoter regions of *flhDC* and *prtA* (500 nt upstream the respective ATG translational start codons) were cloned in the pPROBE::gfp [ASV] plasmid and each reporter construction was transformed into wild-type cells constitutively expressing an mCherry fluorescent marker. We verified that the plasmids-harboring strains displayed an equivalent biofilm growth phenotype to the wild-type one. Images were captured by fluorescence confocal microscopy in a 100 h timeframe, followed by the quantitation of GFP fluorescence levels relative to the values obtained for constitutively expressed m-Cherry (RFP) fluorescence, as described in Materials and Methods. Transcriptional expression levels of *fhlDC* progressively declined from the onset of biofilm formation, showing a 7-fold decrease at 100 h. In contrast, *prtA* transcription peaked at 55 h, showing a fourfold increase relative to the initial levels, followed by a subsequent decline (Fig. 4C and D). Additionally, as a control, the average thickness (µM) and total biomass (µM3/µM2) of biofilm images from both strains, obtained by fluorescence confocal microscopy, were determined (see Fig. S2A and B at http://ibr-conicet.gov.ar/wp-content/uploads/2025/07/Tuttobene-et-al-Supplementary-Material.docx).

These observations reveal an inverse transcriptional pattern of *flhDC* and *prtA* during the establishment of the biofilm. This temporal correlation is consistent with a scenario in which flagellar expression predominates during early adhesion, while *prtA* expression increases during the transition to a sessile lifestyle. This regulation timing suggests that transcriptional and post-translational events may be coordinated during biofilm development. In this context, increased PrtA expression may contribute to the clearance of flagellar components that could otherwise interfere with the maturation and stabilization of the multicellular structure.

**Fig 5:**
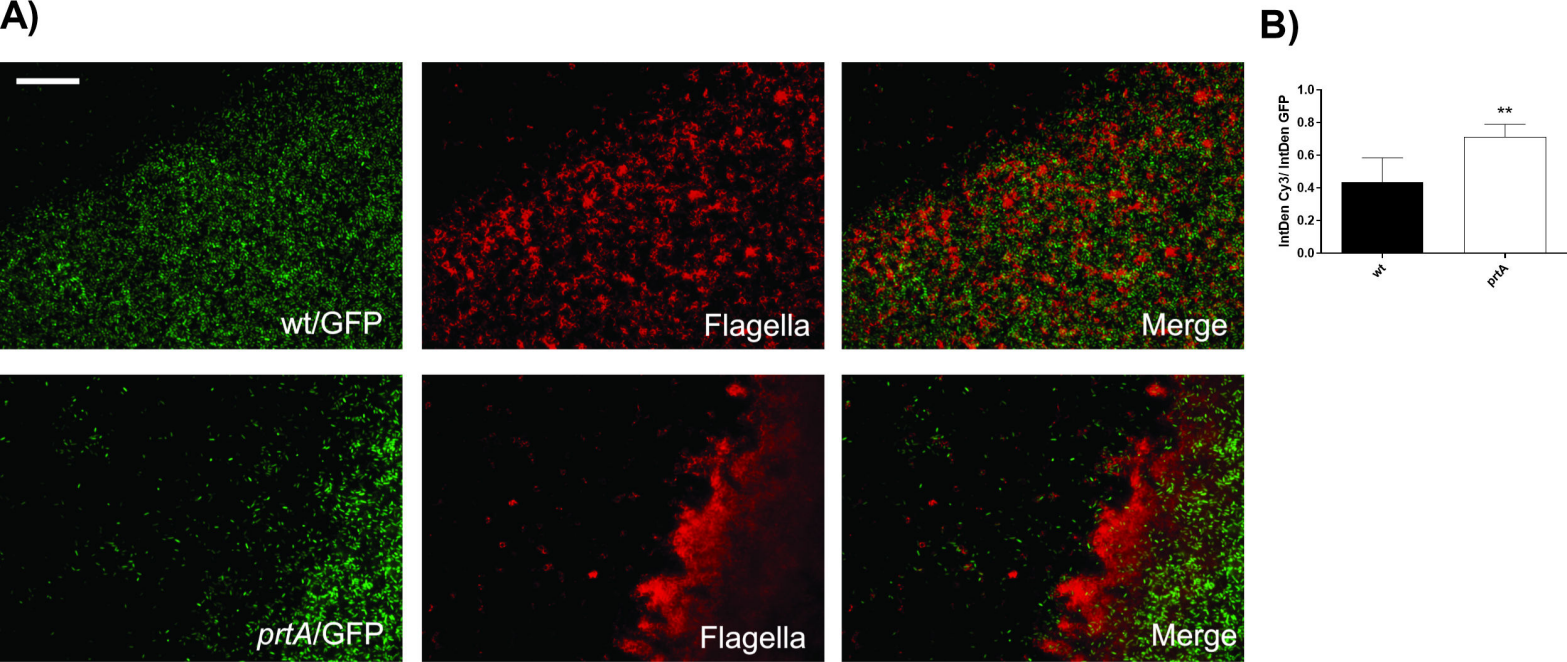
PrtA is required in the removal of flagellin building blocks. (**A**) Micrographs of air-liquid interface biofilm assay on coverslips of wild-type/GFP and *prtA*/GFP strains. Representative images obtained by fluorescence microscopy with a magnitude of magnification of 100 X are shown. (**B**) Quantification of flagellin fluorescence intensity of air-liquid interface biofilm assay on coverslips of wild-type/GFP and *prtA*/GFP strains. Cells were incubated with primary polyclonal antibodies against flagellin (1:100) and detected by incubation with anti‐rabbit Cy3 conjugated secondary antibody (1:150). Flagellin abundance was calculated as a ratio of Cy3 fluorescence and GFP fluorescence (IntDen Cy3/IntDen GFP) of the images obtained by fluorescence microscopy. Significant differences between strains calculated by unpaired *t* test are indicated as follows: *P* < 0.05; ∗∗, *P* < 0.01; ∗∗∗, *P* < 0.001; and ∗∗∗∗, *P* < 0.0001; the analysis was performed using GraphPad Prism (GraphPad Software, San Diego, CA, USA).

We also assayed biofilm formation using the device designed by Merritt et al. (45), which allowed us to capture images by fluorescence confocal microscopy at the liquid-air interface of the developing biofilm. Flagellin was detected in the biofilm matrix of either the wild-type or the *prtA* mutant strains (Fig. 5A). However, *prtA* biofilm showed a 40% increase in flagellin signal compared to the wild-type strain (Fig. 5B). These results reinforce the concept that PrtA is required for the removal of flagellin building blocks, which would otherwise accumulate and hinder the proper assembly of the extracellular matrix network in which bacteria are embedded within the biofilm structure.

## DISCUSSION

Biofilm formation is a critical factor in the pathogenicity and environmental persistence of *S. marcescens*, as it provides protection against antibiotics and enhances colonization in diverse environments (46, 47). This study demonstrates that the extracellular metalloprotease PrtA plays an essential role in this process. The significant reduction in biofilm biomass observed in the *prtA* mutant, and its restoration through co-culture with wild-type cells or by supplementation with purified PrtA, supports the idea that PrtA acts as a secreted factor that modulates the biofilm matrix. This finding aligns with previous reports indicating that secreted proteases contribute to biofilm maturation in other genera (48–50). For instance, in *Pseudomonas aeruginosa* as the zinc metalloproteases LasB and AprA participate in biofilm remodeling and dispersal through degradation of the extracellular matrix components and other secreted proteins (48–50). LasB specifically cleaves structural proteins such as elastin and collagen, while AprA degrades flagellin monomers, helping to clear accumulated debris that may interfere with matrix formation (51). In *Staphylococcus aureus*, the secreted protease aureolysin contributes to biofilm detachment by modulating the accumulation of surface adhesins (52), and in *Bacillus subtilis*, extracellular proteases such as Bpr and NprE have been shown to degrade surface-associated proteins, facilitating matrix remodeling and biofilm dispersion (53–55).

In our study, the catalytic activity of PrtA proved essential for biofilm formation, as a point mutant variant (PrtAE177A) lacking proteolytic activity failed to complement the biofilm-deficient phenotype of the *prtA* mutant. This is consistent with studies showing that bacterial metalloproteases process extracellular matrix components or regulate cell-cell interactions required for biofilm architecture (56, 57). Proteomic analyses of extracellular and matrix-associated fractions revealed significant accumulation of flagellar structural proteins—particularly FliC—in the *prtA* mutant. These results were corroborated by *in vitro* degradation assays showing that PrtA, like AprA in *P. aeruginosa*, degrades depolymerized but not polymeric flagellin (36). The selective degradation of monomeric FliC suggests a role for PrtA in managing flagellar turnover products during biofilm maturation.

Flagella play a dual role in biofilm biology: they are critical for initial surface attachment and motility-driven aggregation but must be downregulated or disassembled during the transition to a sessile lifestyle. This transition is tightly regulated and conserved across many bacterial genera. In *Escherichia coli*, biofilm formation is associated with suppression of the *flhDC* operon, the master regulator of flagellar genes, and upregulation of genes encoding matrix components (58). Our observation that *prtA* expression peaks as *flhDC* declines supports the notion that PrtA contributes to this transition by degrading residual flagellar components. This mechanism may be important for clearing proteins that could otherwise interfere with matrix assembly or cell–cell interactions, a hypothesis supported by similar findings in *Vibrio cholerae*, where flagellar remnants can destabilize mature biofilms (59).

In addition to flagellar components, our proteomic analysis identified other differentially abundant proteins in the *prtA* mutant, including BssS and OmpX. BssS is a small regulatory protein in *E. coli* known to affect biofilm formation by modulating intracellular levels of cyclic-di-GMP, a secondary messenger that controls the transition between motility and sessility (60). Its overaccumulation in the *prtA* mutant may reflect a compensatory response to altered surface or matrix cues. OmpX, a conserved outer membrane protein in enterobacteria, has been linked to adhesion, virulence, and stress adaptation (61). In *Citrobacter werkmanii*, OmpX is essential for biofilm formation, osmotolerance, and motility (62). These parallels suggest that the increased abundance of OmpX in the *prtA* mutant could contribute to altered biofilm architecture or surface interactions, highlighting a broader regulatory role for PrtA in modulating cell envelope composition.

Beyond the degradation of specific structural components, PrtA may serve to prevent the accumulation of extracellular protein aggregates that could obstruct nutrient flow or signaling within the biofilm. Analogous roles have been described for LasB in *P. aeruginosa*, where matrix degradation not only facilitates dispersal but also restores biofilm architecture in response to environmental cues (63). In *B. subtilis*, Bpr and NprE act during biofilm dispersal stages by degrading matrix proteins and enabling cells to return to planktonic growth (64, 65). Interestingly, a study by Selan et al. on *S. marcescens* reported that a catalytically inactive variant of the serralysin-family protease Spep was still able to impair biofilm formation in *Staphylococcus aureus*, suggesting that proteases like PrtA may also exert regulatory effects independent of their enzymatic activity (27). Such effects could include interactions with other surface proteins or modulation of extracellular signaling pathways. It has been postulated that motile bacteria must become non-motile when transitioning to the biofilm state. This transition has been described to take place in two stages: a rapid inhibition of flagellar function and a slow inactivation at the level of flagellar gene expression (66). Numerous signals, including the contact of bacteria with a surface and mechanisms that involve signal transduction systems and multiple transcriptional regulators, have been reported to be involved in this process (67). A considerable amount of effort has been dedicated to determining the stoichiometry and turnover of proteins such as FliM and FliN within functioning flagellar motors and to understanding FlgM turnover that directs critical steps of the flagellar assembly cascade (68). It has also been postulated that flagella are diluted to extinction through growth within the biofilm (59). However, little is known about the fate of flagellar components upon inactivation, disassembly, or ejection of this appendage by shearing forces. The accumulation of flagellar components can be detrimental to biofilm integrity, as they may interfere with the extracellular matrix and cell-cell interactions (59). In *P. aeruginosa*, the metalloprotease AprA, homologous to PrtA, degrades depolymerized flagella, aiding in biofilm formation by modulating the availability of flagellar components (69). Similarly, in *Vibrio vulnificus*, flagellin-homologous proteins interact with exopolysaccharides, essential for biofilm maturation, highlighting the intricate relationship between flagellar elements and biofilm matrix components (70).

**Fig 6:**
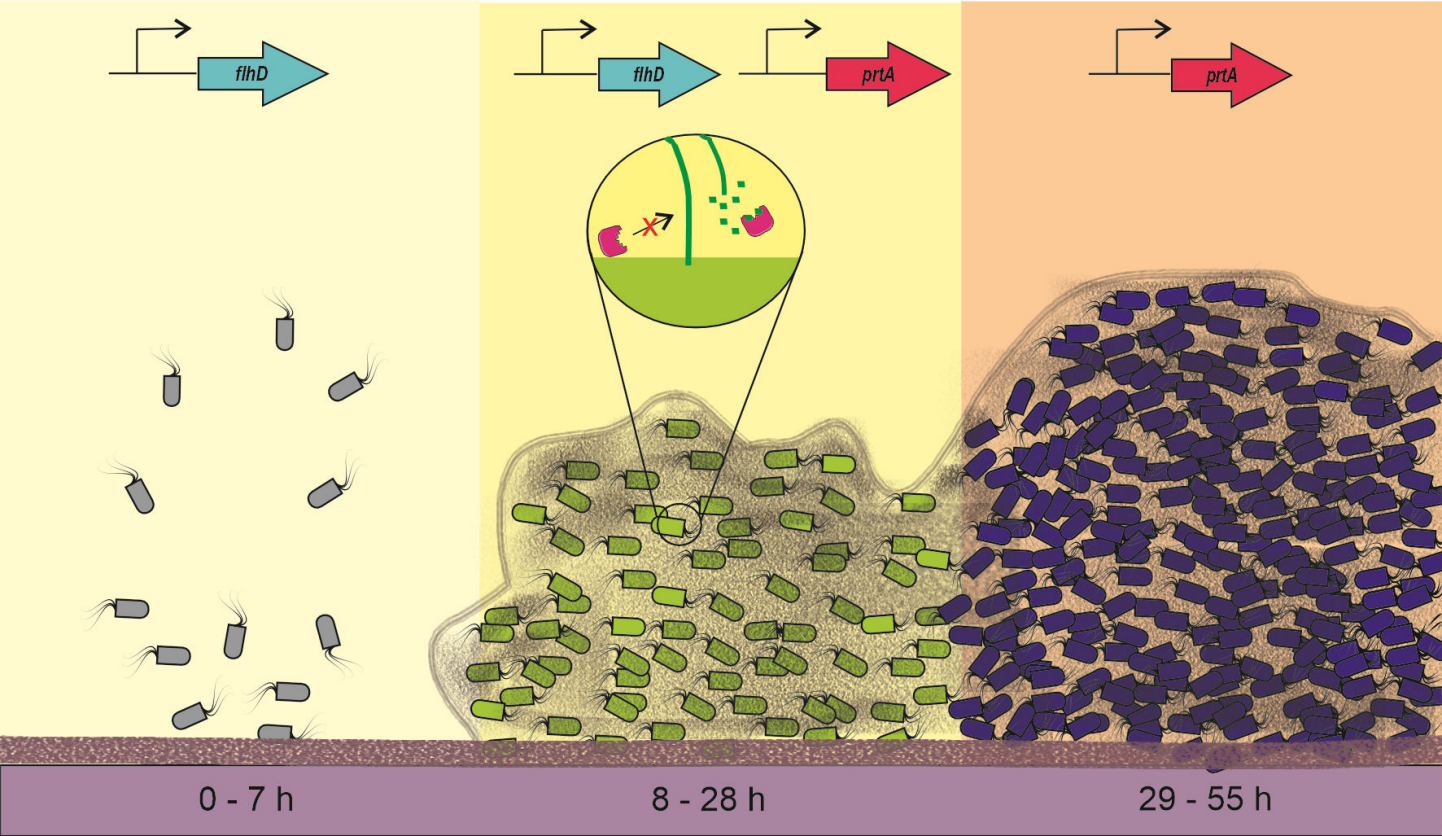
Proposed model for PrtA and the flagellum participation and their gene expression during biofilm formation.

Taken together, our findings support a model in which PrtA coordinates biofilm maturation by targeting not only flagellar remnants but also a broader array of membrane-associated and stress-related proteins. By modulating matrix composition and reducing interference from residual structural elements, PrtA facilitates the transition from motility to sessility. These activities appear to be conserved among diverse bacterial genera, underscoring the importance of secreted proteases in orchestrating the spatial and temporal dynamics of biofilm development. Understanding the precise mechanisms of PrtA function may provide new strategies for biofilm control and disruption in clinical and environmental settings. The proposed model and phenotypes examined in this work are summarized in the scheme shown in Fig. 6.
